# Repeatability of [^15^O]H_2_O PET imaging for lower extremity skeletal muscle perfusion: a test–retest study

**DOI:** 10.1186/s13550-024-01073-x

**Published:** 2024-01-31

**Authors:** Nana Louise Christensen, Jens Sørensen, Kirsten Bouchelouche, Michael Alle Madsen, Christian Selmer Buhl, Lars Poulsen Tolbod

**Affiliations:** 1 Department of Clinical Medicine, Aarhus University, Palle Juul-Jensens Boulevard 165, 8200 Aarhus N, Denmark; 2 Steno Diabetes Center Aarhus, Aarhus University Hospital, Aarhus, Denmark; 3 Department of Nuclear Medicine & PET, Aarhus University Hospital, Aarhus, Denmark

## Abstract

**Background:**

[^15^O]H_2_O PET/CT allows noninvasive quantification of tissue perfusion and can potentially play a future role in the diagnosis and treatment of peripheral artery disease. We aimed to evaluate the reliability of dynamic [^15^O]H_2_O PET imaging for measuring lower extremity skeletal muscle perfusion. Ten healthy participants underwent same-day test–retest study with six dynamic [^15^O]H_2_O PET scans of lower legs and feet. Manual volume-of-interests were drawn in skeletal muscles, and PET time activity curves were extracted. *K*_1_ values (mL/min/100 mL) were estimated using a single-tissue compartment model (1TCM), autoradiography (ARG), and parametric imaging with blood input functions obtained from separate heart scans.

**Results:**

Resting perfusion values in the muscle groups of the lower legs ranged from 1.18 to 5.38 mL/min/100 mL (ARG method). In the muscle groups of the feet, perfusion values ranged from 0.41 to 3.41 mL/min/100 mL (ARG method). Test–retest scans demonstrated a strong correlation and good repeatability for skeletal muscle perfusion with an intraclass correlation coefficient (ICC) of 0.88 and 0.87 and a repeatability coefficient of 34% and 53% for lower legs and feet, respectively. An excellent correlation was demonstrated when comparing volume-of-interest-based methods (1TCM and ARG) (lower legs: ICC = 0.96, feet: ICC = 0.99). Parametric images were in excellent agreement with the volume-of-interest-based ARG method (lower legs: ICC = 0.97, feet: ICC = 0.98).

**Conclusion:**

Parametric images and volume-of-interest-based methods demonstrated comparable resting perfusion values in the lower legs and feet of healthy individuals. The largest variation was seen between individuals, whereas a smaller variation was seen between muscle groups. Repeated measurements of resting blood flow yielded a strong overall correlation for all methods.

**Supplementary Information:**

The online version contains supplementary material available at 10.1186/s13550-024-01073-x.

## Introduction

Peripheral artery disease (PAD) is a vascular condition that affects the arteries supplying blood to the lower limbs, potentially leading to critical limb ischemia (CLI) characterized by rest pain, non-healing wounds, and, in severe cases, requiring amputation. Ensuring precise evaluation of perfusion is of utmost importance in optimizing treatment and clinical outcomes in individuals with PAD [1].

Various imaging techniques, including positron emission tomography (PET) and single photon emission computed tomography (SPECT), have been utilized to measure skeletal muscle perfusion in the lower limbs. In studies conducted by researchers from Ohio and Yale, static SPECT images were used to measure the uptake of [^99m^Tc]-tetrofosmin in the feet 20-min post-injection, enabling the monitoring of disease progression [2] and prediction of amputation risk in CLI patients [3]. Dynamic PET imaging has been used for measuring skeletal muscle perfusion in several studies [4, 5] and may have an advantage over SPECT imaging as quantification of perfusion can be performed. However, few studies have examined perfusion in the lower legs and to our knowledge, no studies of perfusion in feet using PET have been published. Scremin et al. used [^15^O]H_2_O PET to assess perfusion capacity in the lower leg of CLI patients [5]. This approach utilized the autoradiographic method (ARG) [6] with a partial volume-corrected image-derived input function for quantification [7].

In the current study, we investigate the same-day repeatability of quantification of muscle perfusion in lower legs and feet in healthy subjects using [^15^O]H_2_O PET. Quantification was performed using VOI-based modeling with both the ARG method and a standard one-tissue compartment model (1TCM) as well as an image based-approach with parametric mapping of perfusion. Like Scremin et al. [5], we employed a noninvasive approach with an image-derived input function. However, instead of performing partial volume correction, we utilize an input function derived from a separate heart scan [8, 9]. Since PAD patients may exhibit very heterogeneous tissue bolus arrival times, voxel-based delay corrections were performed for parametric images. By assessing the consistency of repeated measurements, the study aimed to offer valuable insights into the reliability of this imaging technique for evaluating skeletal muscle perfusion in the lower extremities. The findings could have significant implications for diagnosing and treating conditions that impact blood flow and tissue perfusion in the lower limbs.

## Methods

### Study population

Ten healthy participants were included in the study. Participants who had a history of vascular surgery, leg pain (including rest pain), smoking or regular use of other nicotine products, were younger than 40 years old or older than 90 years old, pregnant, claustrophobic, severely obese, or previously treated with chemotherapy were excluded. The study was conducted in accordance with the ethical principles outlined in the Declaration of Helsinki and approved by the local ethics committee (Central Denmark Region Committees on Health Research Ethics). Written informed consent was obtained from all participants.

### PET acquisition

**Fig. 1:**
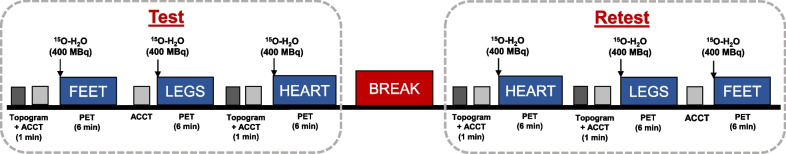
Test–retest imaging protocol for same-day repeatability of [^15^O]H_2_O PET skeletal muscle perfusion in lower extremities

All PET/CT exams were performed on the same day as shown in Fig. 1. Prior to the examinations, participants were instructed to fast for a minimum of 2 h and abstain from caffeine for at least 24 h. Participants were positioned in a supine position on the scanner bed and were instructed to rest for 5 min with their arms in an elevated position. Systolic and diastolic blood pressure and heart rate were then recorded.

Scans were performed using a Siemens Vision PET/CT system (Siemens Healthineers, Knoxville, USA) with a 26 cm axial field of view. The scan protocol consisted of three paired low-dose CT scans and 6-min dynamic list mode PET acquisitions of first the feet, then lower legs and, finally the heart as illustrated in Fig. 1. Simultaneously with the initiation of each PET acquisition, participants were administered a standardized 400 MBq intravenous bolus injection of [^15^O]H_2_O using a MedRad Contrast Infusion pump (10 mL, 1 mL/s), followed by infusion of 30 mL saline. The dynamic PET data of the heart and lower leg scans were divided into 22 time frames (1 × 10 s, 8 × 5 s, 4 × 10 s, 2 × 15 s, 3 × 20 s, 2 × 30 s, 2 × 60 s). The dynamic PET data of the feet scans was divided into 26 time frames (1 × 20 s, 10 × 5 s, 5 × 10 s, 4 × 15 s, 3 × 20 s, 2 × 30 s, 1 × 60 s). Different time frame structures were used for legs and feet to accommodate differences in arrival time. All images were reconstructed to 2.75 × 2.75 × 3 mm^3^ voxels using a time-of-flight ordered-subset expectation maximization algorithm (TrueX, 4 iterations, 5 subsets) with all corrections applied. Following the scan of the heart, the participants were instructed to exit the scanner and engage in a brief walking activity for a duration of 5 min. Following the walking activity, participants were re-positioned on the scanner bed and instructed to rest for an additional 5 min. The three scans were then repeated in reverse order, starting with the heart.

### Data analysis

Volumes of interest (VOI) were drawn manually within the skeletal muscles of the lower legs and feet using PMOD (version 4.006, PMOD Technologies GmbH., Zurich, Switzerland). An overlay of CT images and summed early frames (1–10) of the PET, representing the blood phase of the scan, was used to ensure large blood vessels were not included in the VOIs. The lower leg muscle groups included gastrocnemius (GAS), soleus (SOL), peroneus longus (PL), and tibialis anterior (TA). The muscle groups in the feet included abductor digiti minimi (ADM), abductor hallucis (AH), flexor digitorum brevis (FDB), and flexor hallucis brevis (FHB). PET time activity curves (TAC) were extracted from the VOIs and used for kinetic modeling of tissue perfusion.

#### Blood input functions

**Fig. 2:**
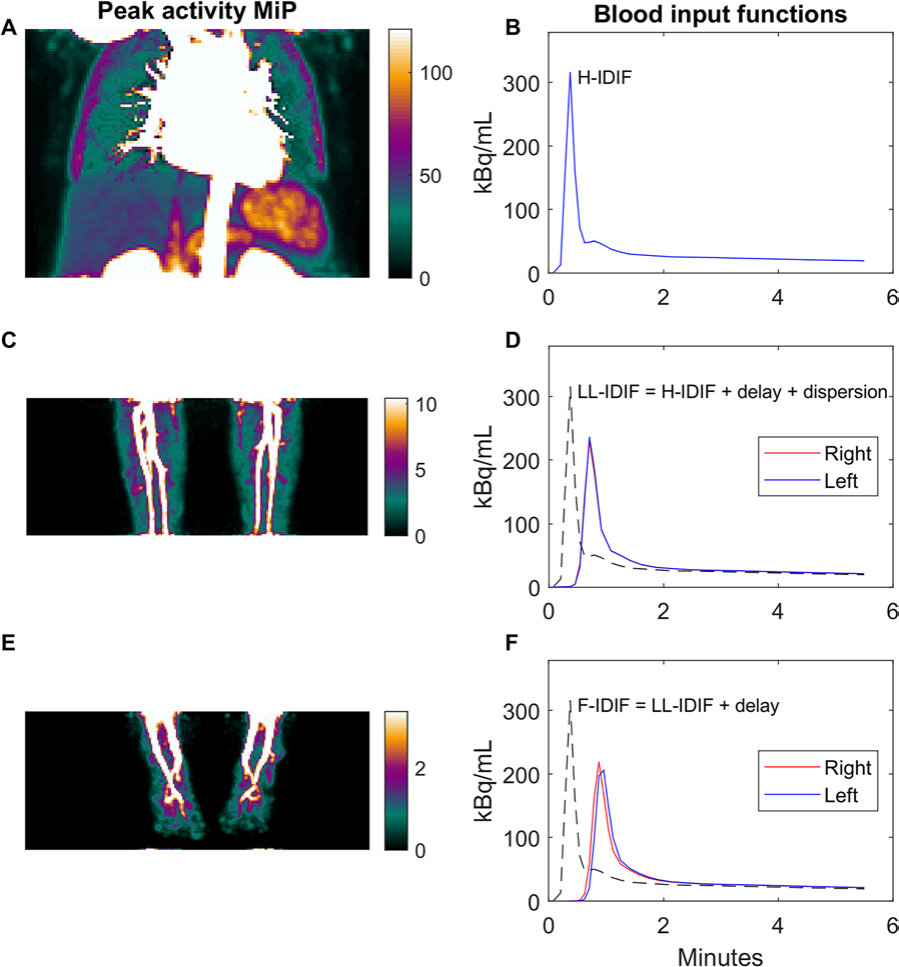
Schematic representation of image-derived input functions (IDIFs) used for the three field of views: heart, lower legs and feet. To the left **A**, **C** and **E**, maximum intensity projections (MIP) of the peak activity to illustrate the arterial signal in the three field of views. To the right, the input functions for the heart (**B**, H-IDIF), lower leg (**D**, LL-IDIF) and feet (**F**, F-IDIF) field of views. The IDIF from the heart field of view (H-IDIF, **A** and **B**) was used to derive IDIFs for all field of views. Delay and dispersion from the heart to each lower leg were estimated from the arterial signal in the corresponding lower leg (**C**) and added to the H-IDIF to yield two LL-IDIFs (left and right, **D**). Similarly, delay from the lower leg to the feet was estimated from the arterial signal in each foot (**E**) (additional dispersion from lower leg to foot was ignored) and added to the LL-IDIFs to yield two F-IDIFs (left and right, **F**). The H-IDIF is shown in dotted lines in **D** and **F** for reference

Blood input functions were obtained from a separate scan of the heart as previously described for quantification of tumor perfusion [9]. Briefly, the [^15^O]H_2_O PET scan of the heart was analyzed using aQuant (MedTrace Pharma A/S, Hørsholm, Denmark) which automatically extracts a TAC for the aorta from the dynamic scan using a cluster method (heart image-derived input function, H-IDIF) [10]. The same cluster method was then used to extract arterial TACs from the scans of the left and right lower leg (LL) and foot. The LL extremity TACs were used to assess delay and dispersion relative to the heart [9, 11], which was then added to H-IDIF to yield the blood input functions for each leg (LL-IDIF left and right, Fig. 2). In the feet, only delay could be robustly estimated, and it was assumed that the same dispersion as for the lower leg could be used. Consequently, IDIFs for each foot (F-IDIF left and right) were calculated by adding a delay to the LL-IDIFs. The repeatability of the IDIF was estimated by comparing the forward cardiac output (calculated as injected activity divided by area under the first-pass curve) [12] from the test and retest scans.

#### VOI-based quantification

Two different modeling approaches were utilized: (1) Compartment modeling with single-tissue compartment (1TCM), and (2) the ARG method. The 1TCM model was fitted to the tissue TACs for each VOI using nonlinear least-squares optimization (in-house software written in MATLAB, MathWorks, Natick, Massachusetts, USA) [10]. The model included an additional delay, d*t*, for each VOI as well as arterial blood volume, *V*_A_. *K*_1_ corresponded to tissue perfusion.

$${C}_{{\text{PET}}}={{C}_{{\text{A}}}\left(t+{\text{d}}t\right)\otimes K}_{1}{e}^{-{k}_{2}t}+{V}_{{\text{A}}}{C}_{{\text{A}}}\left(t+{\text{d}}t\right)$$

Due to the low perfusion in skeletal muscles at rest, *k*_2_ could not be reliably estimated [4]. The ARG method [6] utilizes integrated tissue TACs and perfusion look-up tables generated from the IDIF and a fixed partition coefficient (*λ *= 0.99). The model assumes arterial blood volume can be neglected. Look-up tables were generated using ARLKUP (TCPCLIP 0.6.6, Turku PET Centre, Turku, Finland). A two step-process was used for the ARG method, where delay for the VOI was first estimated using a 1TCM model and then added to the IDIF before generation of the look-up table.

#### Parametric images

Parametric images of perfusion were generated using the ARG method applied at voxel level using in-house software (MATLAB). However, the ARG method does not account for delay, which can vary across different regions of the image. To address this, the raw dynamic PET images underwent voxel-wise delay correction using a leading-edge approach [13] with a 10% of peak activity threshold before generating the parametric images. Additionally, to enhance the robustness of the leading-edge detection and minimize noise, the raw dynamic images were initially subjected to denoising using HYPR-LR (HighlY constrained backPRojection to Local Regions of interest) [14]. For comparison with VOI-based quantification, parametric images were imported to PMOD, and the same VOIs used for extracting TACs were transferred to parametric images, and voxel-mean perfusion was calculated.

### Statistical methods

The agreement between 1TCM, ARG and parametric images as well as the repeatability of IDIFs and skeletal muscle perfusion in lower extremities were assessed by Bland–Altman analysis. Data normality was assessed using Shapiro–Wilk test. Perfusion data were log-transformed, and the within-subject/within-muscle coefficient of variability, repeatability (RPC) and intraclass correlation coefficients (ICCs) was calculated using the formalism described in details by Lodge et al. [15]. In short, the repeatability coefficient was calculated as 1.92 × √2 × wCV, where wCV is the within-subject/within-muscle coefficient of variation calculated for log-transformed data. Bland–Altman plots of perfusion measures are presented in the original scale, whereas limits of agreement are back-transformed using the methodology of Euser et al. [16]. The statistical significance of differences in medians was evaluated using the Wilcoxon signed-rank test for pairwise comparisons and otherwise the Mann–Whitney test. *P* values < 0.05 were considered statistically significant. Sample size calculations were performed using a two-sided significance test of no difference for paired log-normally distributed data with a significance level of 5% and a power of 95%. The sample size calculations were based on the standard deviation of the difference between the logarithm of the test and the logarithm of the retest.

Study data were collected and managed using REDCap (Vanderbilt University Medical Center, Nashville, TN, USA) [17]. Data analysis was performed using Stata version 18.1 (StataCorp LLC, College Station, TX, USA).

## Results

The demographic and physiological characteristics of the study participants are presented in Table 1.

**Table 1:** Participant characteristics

	Test	Retest
Number	*n *= 10	
Male/female	7/3	
Age (years)	53 [41–69]	
Weight (kg)	93 [62–140]	
Body mass index (kg/m^2^)	29 [22–46]	
Systolic blood pressure (mmHg)	114 [88–144]	118 [94–150]
Diastolic blood pressure (mmHg)	68 [52–93]	70 [50–89]
Heart rate (beats/min)*	60 [42–71]	60 [42–74]

Mean values are presented with minimum and maximum values in square brackets

*Measured after resting for at least 5 min in supine position

**Fig. 3:**
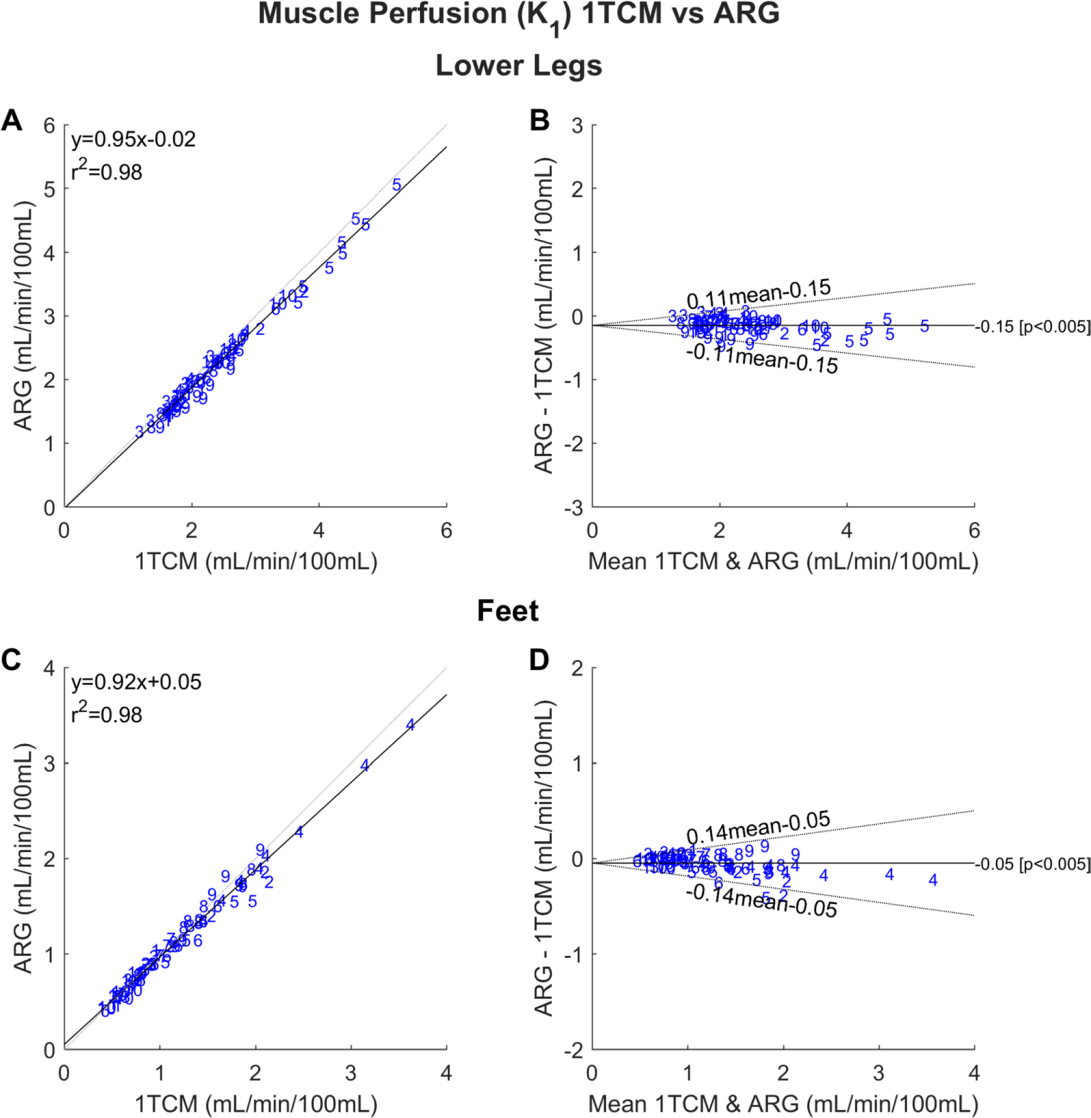
Correlation plots **A** and **C** and Bland–Altman plots **B** and **D** comparing the 1TCM and ARG methods in lower legs **A** and **B** and feet **C** and **D**. The blue numbers represent participant identification. The analysis includes perfusion values from all participants, both the left and right leg and foot (*n *= 80). The correlation plots compare the *K*_1_ values estimated from the methods and include coefficient of determination (*r*^2^) and the linear equation. The dashed lines represent the lines of identity, while the solid lines represent the linear fit. The Bland–Altman plots display the mean difference between the *K*_1_ measurements from the ARG and 1TCM method, with the dashed lines representing the 95% upper and lower limits of agreement

**Fig. 4:**
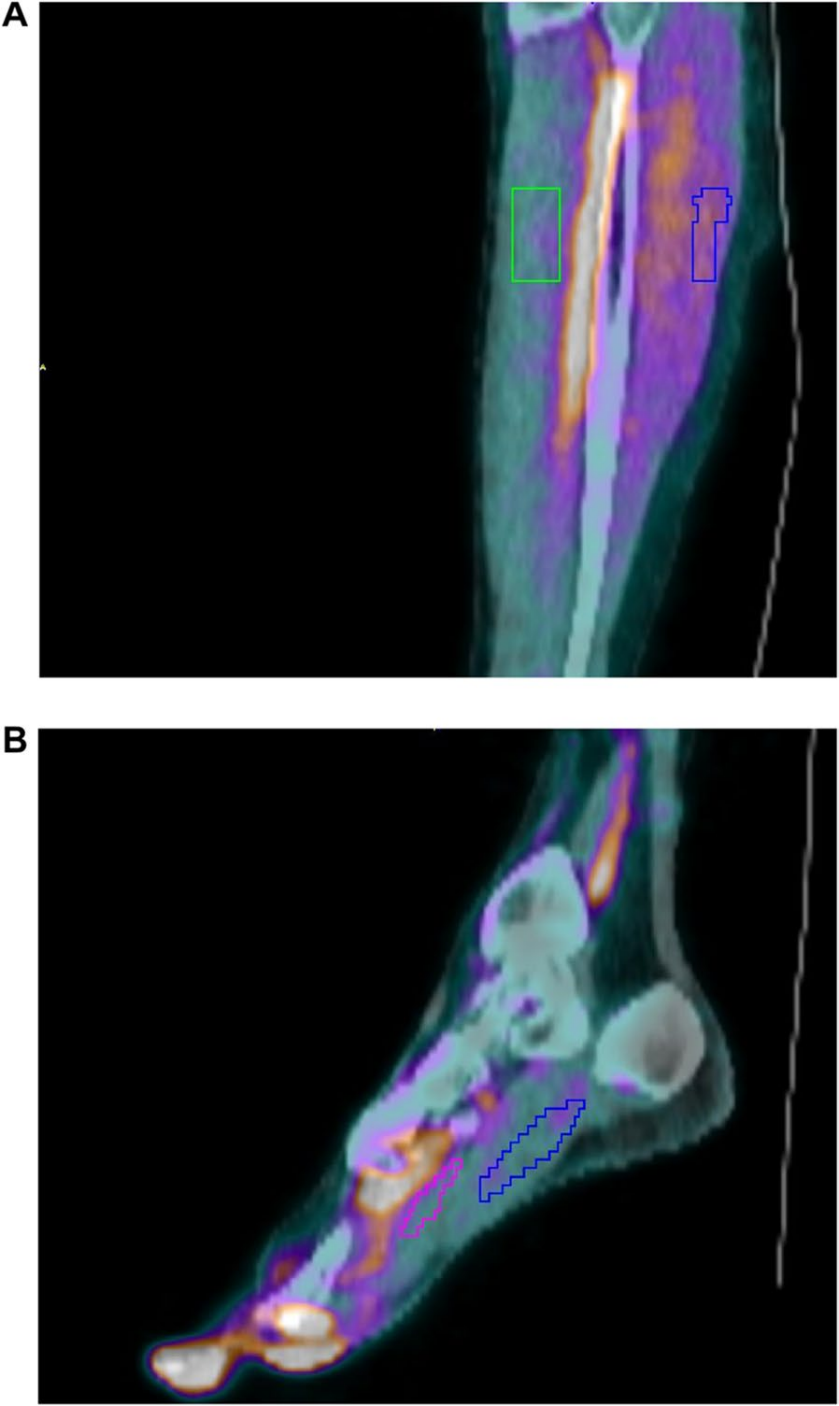
Example of parametric images of tissue perfusion (sagittal view) overlayed on CT. **A** Right lower leg with VOIs for GAS (green) and TA (blue). Color scale ranges from 0 to 6 mL/min/100mL. **B** Right foot with VOIs for FDB (pink) and AH (blue). Color scale ranges from 0 to 4 mL/min/100mL. Since the ARG method does not include blood volume correction, blood vessels and tissue with high blood volume appear as high perfusion

Excellent agreement was found between all three methods. For both lower legs and feet, the 1TCM and ARG method resulted in linear correlations with slopes close to unity, *r*^2^ = 0.98 (Fig. 3A and C) and ICC of 0.96 and 0.99 (Table 2), respectively. Bland–Altman plots (Fig. 3B and D) revealed negligible bias. The agreement between the VOI-based ARG method and the parametric maps was similarly excellent with slopes again close to unity (Additional file 1: Fig. S2) and *r*^2^ > 0.98 and ICC > 0.97. An example parametric *K*_1_ map is shown in Fig. 4. A small positive bias was found for lower legs (0.17 mL/min/100mL), mainly due to an overestimation in a single subject (subject number 5).

**Table 2:** Test–retest statistics

	ICC	RPC (%)	ICC	RPC (%)
	Lower legs		Feet	
1TCM	0.87	35	0.83	66
ARG	0.88	34	0.87	53
Param	0.86	38	0.90	46
1TCM versus ARG	0.96	–	0.99	–
ARG versus Param	0.97	–	0.98	–

*ICC* Intraclass coefficient, *RPC* Repeatability coefficient, *1TCM* Single-tissue compartment model, *ARG* Autoradiography, *Param* Parametric images using the ARG method

Resting normal perfusion values in muscle groups of the lower legs using the ARG method ranged from 1.19 to 5.07 mL/min/100 mL (median 1.98, IQR 0.85). When utilizing the 1TCM method, the resting perfusion values in the lower legs ranged from 1.18 to 5.21 mL/min/100 mL (median 2.13, IQR 0.87). For the muscle groups of the feet, the resting perfusion values using the ARG method ranged from 0.41 to 3.41 mL/min/100 mL (median 0.99, IQR 0.73), while with the 1TCM method, the range was 0.43 to 3.63 mL/min/100 mL (median 1.03, IQR 0.78). The median perfusion values for the individual muscle groups from the test and retest scans are presented in Table 3. There were no differences at muscle level between test and retest, and the only statistically significant difference between muscles in the left and right leg was seen in GAS (*p *= 0.02). The perfusion values of the test and retest scans from all participants can be found in Additional file 1: Tables S1 and S2. The data include perfusion values for all the included muscle groups in the lower legs and feet from both the left and right leg. Arterial blood volume determined using 1TCM was below 1% in all examined muscles. There were no correlations between systolic or diastolic blood pressure and muscle perfusion.

**Table 3:** Resting perfusion, *K*_1_, (mL/min/100mL) in muscle groups of the lower legs and feet

	ARG			
Muscle group	Test (*L*)	Test (*R*)	Retest (*L*)	Retest (*R*)
*Lower legs*				
GAS	2.05 [0.79]	2.23 [0.84]*	1.98 [0.83]	2.05 [0.56]
SOL	2.43 [0.82]	2.57 [1.18]	2.61 [0.77]	2.38 [1.12]
TA	1.74 [0.53]	1.74 [0.36]	1.73 [0.34]	1.68 [0.35]
PL	1.63 [0.83]	1.93 [0.71]	1.75 [0.67]	1.93 [0.46]
*Feet*				
ADM	1.01 [1.17]	0.83 [0.73]	0.89 [1.37]	0.95 [1.04]
AH	0.99 [0.57]	1.04 [0.45]	1.12 [0.59]	0.93 [0.65]
FDB	1.54 [0.95]	1.07 [0.84]	1.19 [0.63]	1.11 [0.97]
FHB	0.95 [0.69]	0.84 [0.40]	1.07 [1.05]	0.91 [0.67]

Lower extremity resting perfusion values from the test and retest scans. The table displays the median and in square brackets, the IQR of perfusion in different muscle groups of the lower legs and feet in ten healthy participants. Statistically significant difference between perfusion in GAS between left and right leg is marked with asterisk (*)

*ARG* Autoradiography method, *GAS* Gastrocnemius, *SOL* Soleus, *TA* Tibialis anterior, *PL* Peroneus longus, *ADM* Abductor digiti minimi, *AH* Abductor hallucis, *FDB* Flexor digitorum brevis, *FHB* Flexor hallucis brevis, *L* Left, *R* Right

The forward cardiac output determined from the area under the first passage IDIF curve had a good repeatability with *r*^2^ = 0.94, ICC = 0.98, no significant bias and repeatability coefficient (RPC) of 15% for the lower legs (Additional file 1: Fig. S1), suggesting that the IDIF did not change much between test and retest.

**Fig. 5:**
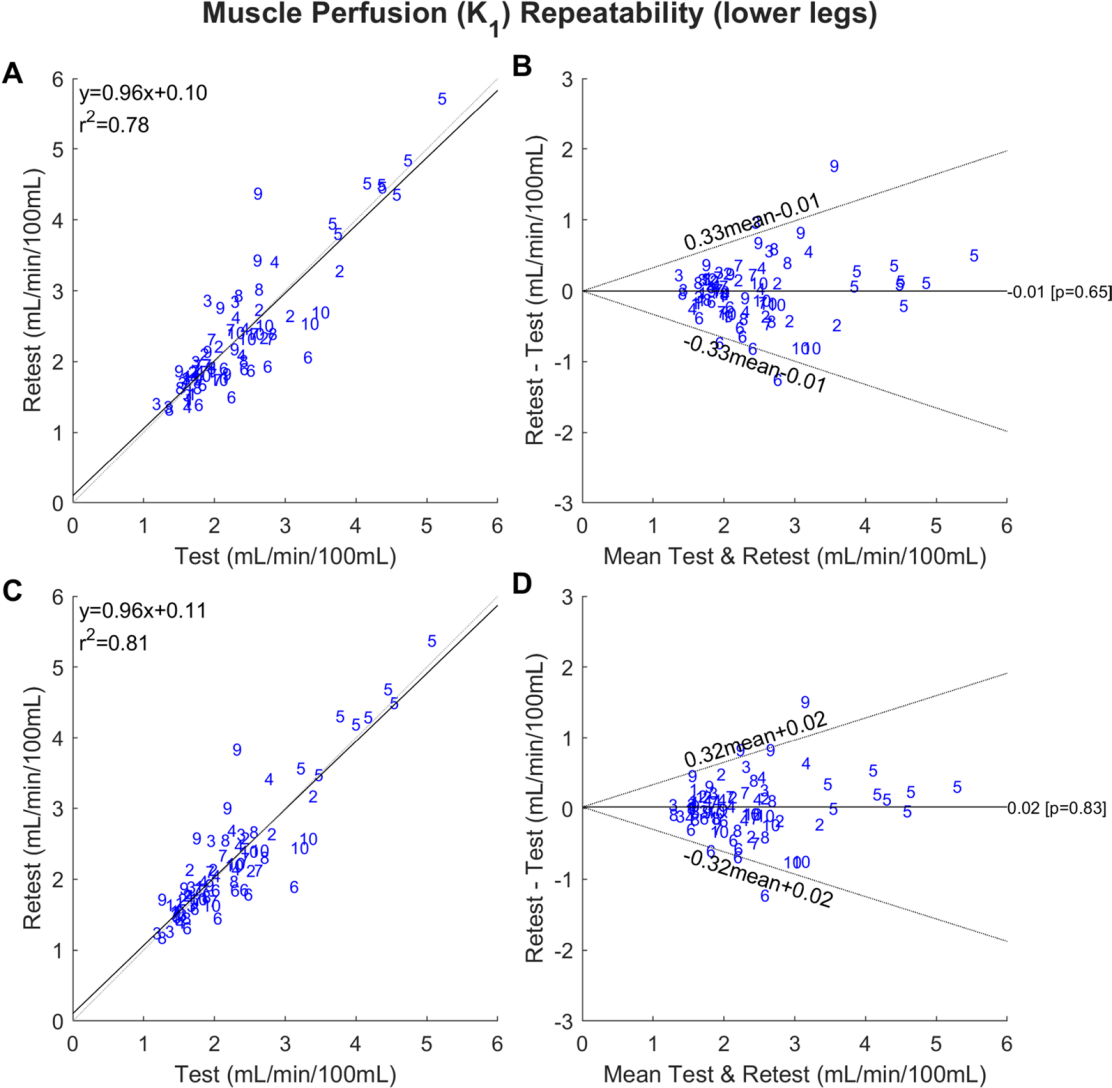
Correlation plots **A** and **C** and Bland–Altman plots **B** and **D** demonstrating the repeatability of *K*_1_ as a measure of skeletal muscle perfusion in the lower legs using the 1TCM (**A** and **B**) and ARG (**C** and **D**). The blue numbers represent participant identification. The analysis includes perfusion values from all participants, both the left and right leg (*n *= 80). The correlation plots compare the *K*_1_ values from the test and retest scan and include coefficient of determination (*r*^2^) and the linear equation. The dashed lines represent the lines of identity, while the solid lines represent the linear fit. The Bland–Altman plots display the mean difference between the *K*_1_ measurements from the retest and test scan, with the dashed lines representing the 95% upper and lower limits of agreement

**Fig. 6:**
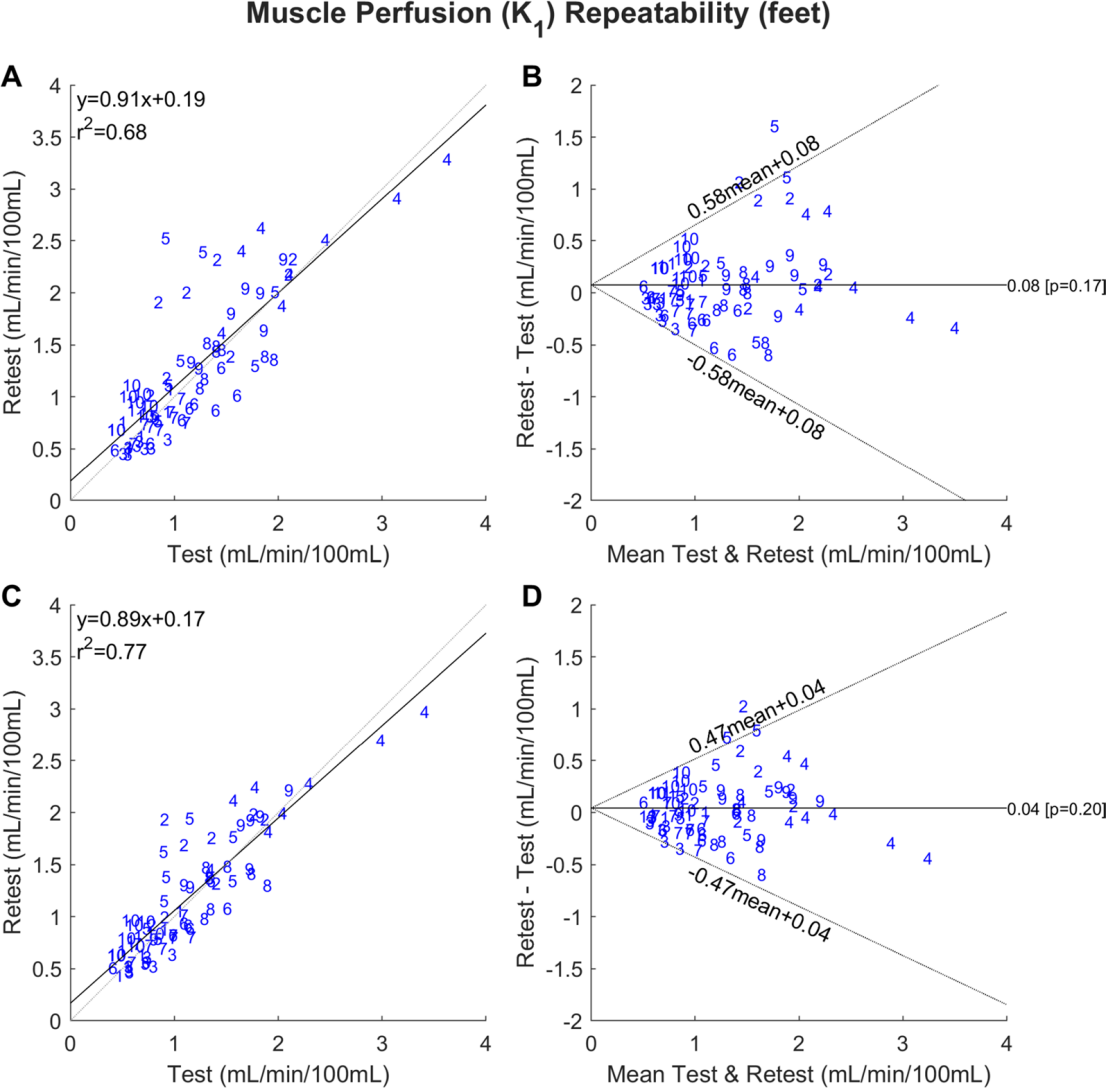
Correlation plots (**A** and **C**) and Bland–Altman plots (**B** and **D**) demonstrating the repeatability of *K*_1_ as a measure of skeletal muscle perfusion in feet using the 1TCM (**A** and **B**) and ARG (**C** and **D**). The blue numbers represent participant identification. The analysis includes perfusion values from all participants, both the left and right foot (*n *= 80). The correlation plots compare the *K*_1_ values from the test and retest scan and include coefficient of determination (*r*^2^) and the linear equation. The dashed lines represent the lines of identity, while the solid lines represent the linear fit. The Bland–Altman plots display the mean difference between the *K*_1_ measurements from the retest and test scan, with the dashed lines representing the 95% upper and lower limits of agreement

The descriptive test–retest statistics of lower extremity muscle perfusion measured by the 1TCM, ARG and parametric images are presented in Table 2. In the lower legs, ICC and RPC were in the range 0.86–0.88 and 34–38%, respectively. The difference between the methods was quite small. Linear correlations and Bland–Altman plots are shown in Fig. 5. Using the ratio of perfusion between the respective muscles in left and right leg (interleg perfusion ratio) or an intraleg perfusion ratio (muscle normalized by the other muscles in same leg) resulted in an improvement in the RPC (RPC 25% and 29%, respectively, Additional file 1: Table S3). In the feet, the repeatability of perfusion was more problematic with ICC ranging from 0.83 to 0.90 and RPC ranging from 46 to 66%. The linear correlations and Bland–Altman for 1TCM and ARG are shown in Fig. 6 and Additional file 1: Fig. S3 for parametric maps. The ARG and parametric methods had the lowest RPCs. In the feet, only using an intrafoot perfusion ratio reduced the RPC, whereas the interfoot perfusion ratio increased it (RPC 47% and 55%, respectively, Additional file 1: Table S3).

## Discussion

Imaging perfusion in lower extremities is of potential interest in PAD and CLI patients as it can be used to quantify regional perfusion and several imaging modalities have been applied including MRI, SPECT and PET [18]. However, clinical application depends on ease-of-use and robustness of the imaging technique. Semi-quantitative SPECT based on SUV (standardized uptake values) fulfills these criteria and was used by Chou et al. [2, 3] on both lower legs and feet with promising results. However, imaging with short-lived PET-tracers may have advantages such as absolute quantification and the possibility to repeat measurements in the same session. This means exercise and other perturbation reserves can be quantified. In addition, the exams are fast (scan time in this study was 6 min per field of view) and the radiation exposure limited (< 2.5 mSv in total for scan of heart, lower leg and feet including CT for localization and attenuation).

In the 90s, two studies utilized [^15^O]H_2_O PET to measure exercise perfusion reserve in PAD patients [4, 19]. Both studies used invasive blood sampling from either a brachial or femoral artery to obtain the blood input function for kinetic modeling. Burchert et al. suggested the utility of semi-quantitative SUV as a proxy for absolute perfusion to avoid arterial blood sampling. Later, Scremin et al. studied perfusion in CLI patients using [^15^O]H_2_O PET avoiding arterial blood sampling by using an image-derived input function from the popliteal artery [5]. Due to the small size of the artery, partial volume correction had to be performed using a measurement of the diameter of the artery from an MRI or contrast CT scan assuming spill-in from the surrounding muscle could be ignored.

In the current study, the problem of intensity recovery and signal spill-in was solved by using an IDIF obtained from a separate scan of the heart. This approach has previously been validated for imaging of tumor perfusion in the pelvis [9].

### Resting perfusion normal values

Using heart-IDIF method and ARG, perfusion in the muscles of the lower leg (median 1.98, range 1.19–5.07 mL/min/100 mL) was comparable to previous PET studies [4, 5]. One subject stood out and had perfusion greater than 3 mL/min/100mL in all muscles. This was likely due to physical exercise performed earlier on the scan day (cycled to the hospital). Perfusion in GAS muscle was slightly higher in the right leg (Table 3, 2.23 vs. 2.05 mL/min/100 mL, *p *= 0.02), but this was the only significant difference between leg at muscle level. However, perfusion was markedly higher in large posterior muscles (GAS and SOL) compared to TA and PL (median 2.31 vs. 1.76 mL/min/100 mL, *p *= 0.001). This is also apparent in the parametric map in Fig. 4. Similarly, the perfusion in the SOL muscle was higher than in the GAS muscle (median 2.46 vs. 2.17 mL/min/100 mL, *p *< 0.001). Perfusion in the muscles of the feet was found to be slightly lower than in muscles of the lower legs (median 1.03, range 0.43–3.63 mL/min/100 mL). To our knowledge, perfusion in feet has not previously been quantified with PET, and only a few studies using MRI or CT have been published. Arterial spin labeling MRI appears to yield perfusion approximately 10 times higher than the current study in both lower legs and feet [20–22], whereas a small dynamic CT study in patients with diabetic foot found perfusion values more in line with the current study (0.5–0.7 mL/min/100 mL in healthy tissue of the feet) [23]. In both lower legs and feet, the interindividual variation was larger than the intraindividual variation in muscle perfusion (coefficient of variation 37% vs. 29% and 71% vs. 40%, respectively).

### 1TCM versus ARG

The ARG method has been widely used for quantification of perfusion in skeletal muscles [5, 6, 24]. It assumes a 1TCM with all parameters except *K*_1_ fixed such that an integrated signal can be used instead of the whole TAC. This makes the method less sensitive to noise, which is especially useful for tissue with low perfusion. However, fitting of a 1TCM model to the tissue TACs is a more flexible and allows incorporation of delay and blood volume as parameters of the model. The high flexibility comes at a cost of potentially less stability of the parameters. Nevertheless, the agreement between 1TCM and ARG in the current study was excellent with ICC greater than 0.96, narrow limits of agreement in both legs and feet (Fig. 3) and only slightly lower perfusion values in the ARG method (bias − 0.05 to − 0.15 mL/min/100 mL). This means the two methods can be used interchangeably which may be advantageous if the assumption of negligible blood volume cannot be fulfilled. The latter could be the case in diabetic foot ulcers or PAD. It is worth noting that muscle groups exhibit similar significant differences when analyzed using the 1TCM method, as they do with the ARG method, suggesting that blood volume contribution is not a factor in these observed differences.

### Parametric imaging

Parametric mapping of perfusion is an attractive alternative to VOI-based quantification. Images can be generated in connection with the scan session and subsequently reviewed in standard PET viewers as a part of a clinical workflow. Parametric images of skeletal muscle perfusion have mainly been generated using the ARG method [5, 24], where the area under the TAC for each voxel is calculated and translated to perfusion units using a look-up table. However, in the lower extremities, anterior and posterior muscles are supplied by different arteries potentially causing differences in delay. In PAD patients, differences in delays could potentially be quite large, just as formation of collateral arteries may play a role. This means a single blood input function delay for the whole field of view cannot be used and voxel-wise delay correction should be performed. To overcome this problem, the current study applied a pulse timing approach [13] to delay correct images prior to formation of parametric images. Parametric images of subject #6 is shown in Fig. 4 with overlayed VOIs. In the lower leg, perfusion is higher posteriorly than anteriorly, whereas in the foot, high perfusion is seen in the toes. In both lower leg and foot, vessels are seen as areas with high perfusion due to the absence of blood volume correction. The agreement between perfusion values obtained from the ARG VOI-based method and ARG parametric images was excellent (ICC > 0.97) with a slight positive bias (0.17 mL/min/100 mL) for the parametric images in the lower leg. Parametric images might be useful for studying tissue heterogeneity and can be used in combination with VOI-based methods to elucidate blood volume contributions.

### Repeatability

The repeatability of perfusion in the muscles of the lower leg was markedly better than in the feet. Using the ARG method, the repeatability was 34% and 53%, respectively. The repeatability in the lower legs is comparable to the repeatability of 37% for tumor perfusion (thorax/abdomen) using [^15^O]H_2_O PET [15]. In the lower leg, the ARG, 1TCM and parametric image methods resulted in similar repeatability. However, in the feet, 1TCM had a markedly higher repeatability coefficient (RPC = 66%). The area under the curve for the IDIF (expressed in units of cardiac output) had a much smaller repeatability coefficient (RPC 15% for lower leg) suggesting that the IDIF method had little contribution on the overall repeatability.

For monitoring treatment response, this means the change from before to after treatment should be at least 34% in the lower leg or 53% in the feet to imply a treatment effect on an individual level. Data on treatment effects on resting muscle perfusion are limited. Chou et al. [3] found differences in resting perfusion in the feet of 3–20% in PAD patients scanned pre- and post-revascularization using SUV-SPECT. However, large VOIs dividing the whole foot into five regions including large vessels and bone were used, making direct comparison difficult. Recent studies using dynamic contrast-enhanced CT found increases in tissue perfusion in the feet of 44–140% post revascularization [25, 26].

In the lower legs, the sample sizes required to detect mean increases of 25%, 50%, and 75% in perfusion were estimated to be 10, 5, and 4, respectively, with a power of 95%. In the feet, the corresponding sample sizes were estimated to be 18, 7, and 5 (Additional file 1: Table S4).

It is worth noting that the repeatability coefficients were slightly higher than the intraindividual variation in perfusion between muscles (29% and 40% for lower legs and feet, respectively), but smaller than the interindividual variation (37% and 70% for lower legs and feet, respectively). Consequently, it may be beneficial to use tissue ratios when comparing individuals, e.g., using the corresponding muscle in the other leg/foot as reference (interleg/foot perfusion ratio) if disease is unilateral, or between muscles in the same leg/foot (intraleg/foot perfusion ratio) if the disease is localized to one angiosome/region. In addition, using an intraleg or foot perfusion ratio improved repeatability (RPC Lower Leg 29% vs. 34%, Feet 47% vs. 53%, Table 2 and Additional file 1: Table S3). On the other hand, using a left–right ratio was only beneficial in the legs and not in the feet (RPC Lower Leg 25% vs. 34%, Feet 55% vs. 53%. Table 2 and Additional file 1: Table S3).

### Limitations

The current study was performed on healthy volunteers at resting conditions and does not examine the repeatability of measuring post-exercise or stress perfusion. Failure to standardize or vary response to the exercise or stress may result in larger repeatability coefficients than the current study. In addition, using a multi-scan-position approach with a short axial field of view scanner may induce variability due to different conditions during the scans just as serial pharmacological stress may not be feasible. To address these issues, tracers that allow for delayed imaging, like ^82^Rb or [^99m^Tc]-tetrofosmin, could be used. However, this approach has limitations in dynamic analysis and absolute quantification. The study was designed as a same-day test–retest study and does not include additional variation that could occur from imaging with longer intervals. This might be relevant in patients with PAD or diabetic foot ulcers since the influence of collaterals, shunting, and inflammation may affect repeatability. Finally, in the current study, VOIs were drawn manually which may have resulted in additional variation which could have been reduced by registration between test and retest images.

### Future perspectives

[^15^O]H_2_O PET is a relatively fast and gentle method for quantifying tissue perfusion in the lower extremities and can, as shown in the current study, be used to create reproducible parametric maps of tissue perfusion even in the feet. The method is promising for studying both resting perfusion and the effect of perfusion perturbation in patients with PAD with relatively low sample sizes needed. In particular, the correlation of tissue perfusion between current diagnostic parameters and outcomes are of interest, but also connection between PAD and heart disease may be pertinent [27].

With the recent advent of large axial field of view PET/CT scanners, the entire leg or even total body can be imaged in one session. This would reduce the number of scans needed and reduce the radiation burden and allow for post-exercise or stress imaging. The presented methods for parametric image formation are directly applicable for these types of scanners, and the blood input function delay correction method may be useful for other tracers as well. The higher sensitivity of large-FOV scanners will allow higher resolution which may reduce signal mixing from different tissues. This may enable regional or even voxel-wise correction of dispersion of the blood input function in addition to delay. The current ARG method was selected due to its lower sensitivity to dispersion [6], but the basis function method [28], which has been used in the heart [10] and pelvis [9] and has the advantage that blood volume can be modeled, may be able to create images with better separation of tissue and blood vessels.

## Conclusion

In healthy individuals, the present study demonstrates good repeatability and substantial positive correlations between the test and retest scans for both VOI-based methods and parametric images in assessing lower leg skeletal muscle perfusion. The greatest variation was observed between individuals, with a lesser variation seen between muscle groups. When utilizing resting perfusion of the lower extremities for evaluation of vascular diseases, such as diabetes, or treatment response, it is important to consider the inter-patient variation in perfusion as well as intra-patient variation induced by the small differences in resting conditions.

### Supplementary Information

**Additional file 1. **Tables of perfusion values (mL/min/100mL) for all muscles, repeatability of intra- and inter leg/foot perfusion ratios, and examples of sample size estimations. Correlation and Bland-Altman plots of test-retest image derived input function, ARG VOI vs parametric images, and test-retest of parametric images in the lower legs.

## Data Availability

The datasets used in this study are available upon reasonable request from the corresponding author.

## Abbreviations

1TCM: One-tissue compartment model
ARG: Autoradiography
CT: Computed tomography
CLI: Critical limb ischemia
ICC: Intraclass correlation coefficient
IDIF: Image-derived input function
MRI: Magnetic resonance imaging
PAD: Peripheral artery disease
PET: Positron emission tomography
RPC: Repeatability coefficient
SPECT: Single Photon emission tomography
TAC: Time activity curve
VOI: Volume of interest
