# Supplementary material for: Repeatability of [15O]H2O PET imaging for lower extremity skeletal muscle perfusion: a test–retest study
Source: EJNMMI Res. 2024 Jan 31;14:11. doi: 10.1186/s13550-024-01073-x (PMC10830956; doi:10.1186/s13550-024-01073-x)
Supplement: Supplementary file 1 — Additional file 1. Tables of perfusion values (mL/min/100mL) for all muscles, repeatability of intra- and inter leg/foot perfusion ratios, and examples of sample size estimations. Correlation and Bland-Altman plots of test-retest image derived input function, ARG VOI vs parametric images, and test-retest of parametric images in the lower legs. [file 13550_2024_1073_MOESM1_ESM.docx]

**S-Table 1: Perfusion values, K_1_ (mL/min/100mL) for test and retest scan of lower legs and feet of all participants using 1TCM**

|  |  | Test | | | |  | Retest | | | | |
| --- | --- | --- | --- | --- | --- | --- | --- | --- | --- | --- | --- |
| Lower legs | | | | | | | | | | | |
| Participant no. | Side | GAS | SOL | TA | PL | | GAS | SOL | TA | PL |  |
| 1 | L | 1.64 | 1.94 | 1.61 | 1.62 | | 1.62 | 1.87 | 1.44 | 1.80 |  |
|  | R | 1.64 | 2.14 | 1.68 | 1.59 | | 1.48 | 1.78 | 1.55 | 1.75 |  |
| 2 | L | 2.63 | 3.07 | 1.69 | 1.86 | | 2.74 | 2.65 | 1.86 | 2.11 |  |
|  | R | 2.70 | 3.77 | 1.73 | 2.06 | | 2.34 | 3.29 | 1.87 | 2.22 |  |
| 3 | L | 1.60 | 2.29 | 1.35 | 1.18 | | 1.77 | 2.85 | 1.37 | 1.41 |  |
|  | R | 1.74 | 1.90 | 1.55 | 1.62 | | 2.00 | 2.87 | 1.71 | 1.53 |  |
| 4 | L | 1.72 | 2.30 | 2.44 | 2.39 | | 1.78 | 2.63 | 2.47 | 2.10 |  |
|  | R | 1.62 | 1.98 | 2.86 | 1.79 | | 1.37 | 1.93 | 3.41 | 1.86 |  |
| 5 | L | 4.37 | 4.57 | 3.74 | 4.15 | | 4.47 | 4.37 | 3.81 | 4.52 |  |
|  | R | 4.36 | 5.21 | 3.67 | 4.72 | | 4.50 | 5.72 | 3.95 | 4.85 |  |
| 6 | L | 2.42 | 2.75 | 2.13 | 1.78 | | 1.90 | 1.94 | 1.91 | 1.40 |  |
|  | R | 2.52 | 3.32 | 1.83 | 2.24 | | 1.88 | 2.07 | 1.68 | 1.50 |  |
| 7 | L | 1.96 | 2.05 | 1.90 | 1.75 | | 2.32 | 1.75 | 1.96 | 1.95 |  |
|  | R | 2.23 | 2.56 | 1.85 | 2.79 | | 2.46 | 2.40 | 1.87 | 2.33 |  |
| 8 | L | 2.34 | 2.63 | 1.36 | 1.51 | | 2.94 | 3.03 | 1.33 | 1.63 |  |
|  | R | 2.41 | 2.82 | 1.76 | 1.96 | | 2.02 | 2.40 | 1.64 | 1.92 |  |
| 9 | L | 1.50 | 2.08 | 2.18 | 1.76 | | 1.87 | 2.76 | 1.83 | 1.76 |  |
|  | R | 2.61 | 2.61 | 2.28 | 1.90 | | 3.44 | 4.38 | 2.19 | 2.15 |  |
| 10 | L | 2.58 | 3.50 | 1.82 | 2.30 | | 2.40 | 2.70 | 1.81 | 2.41 |  |
|  | R | 2.71 | 3.35 | 2.07 | 2.47 | | 2.52 | 2.55 | 1.75 | 2.33 |  |
| Feet | | | | | | | | | | | |
|  |  | ADM | AH | FDB | FHB | | ADM | AH | FDB | FHB |  |
| 1 | L | 0.50 | 0.56 | 0.59 | 0.55 | | 0.76 | 0.52 | 0.88 | 0.48 |  |
|  | R | 0.70 | 0.93 | 0.95 | 0.67 | | 0.85 | 0.86 | 1.08 | 0.63 |  |
| 2 | L | 2.10 | 1.54 | 2.14 | 1.11 | | 2.18 | 1.39 | 2.33 | 2.01 |  |
|  | R | 1.41 | 0.92 | 0.85 | 0.77 | | 2.32 | 1.18 | 1.92 | 1.02 |  |
| 3 | L | 0.55 | 0.55 | 0.51 | 0.66 | | 0.52 | 0.45 | 0.46 | 0.58 |  |
|  | R | 0.64 | 0.94 | 0.71 | 0.78 | | 0.54 | 0.59 | 0.51 | 0.51 |  |
| 4 | L | 3.63 | 1.84 | 2.04 | 1.65 | | 3.29 | 2.63 | 1.88 | 2.41 |  |
|  | R | 3.14 | 1.46 | 2.11 | 2.46 | | 2.91 | 1.62 | 2.18 | 2.51 |  |
| 5 | L | 1.97 | 1.06 | 1.78 | 1.27 | | 2.01 | 1.35 | 1.31 | 2.40 |  |
|  | R | 0.91 | 0.84 | 0.94 | 0.80 | | 2.53 | 0.77 | 1.11 | 0.82 |  |
| 6 | L | 0.77 | 1.07 | 1.60 | 1.45 | | 0.56 | 0.78 | 1.02 | 1.28 |  |
|  | R | 0.43 | 1.19 | 1.40 | 1.15 | | 0.49 | 0.93 | 0.88 | 0.89 |  |
| 7 | L | 0.61 | 1.00 | 0.97 | 0.72 | | 0.55 | 0.80 | 0.86 | 0.74 |  |
|  | R | 0.77 | 1.12 | 1.07 | 0.85 | | 0.72 | 0.76 | 0.99 | 0.69 |  |
| 8 | L | 1.24 | 1.32 | 1.96 | 1.40 | | 1.09 | 1.52 | 1.36 | 1.49 |  |
|  | R | 1.29 | 1.46 | 1.87 | 1.40 | | 1.17 | 1.46 | 1.39 | 1.44 |  |
| 9 | L | 1.82 | 1.24 | 1.86 | 0.82 | | 2.00 | 1.28 | 1.65 | 0.80 |  |
|  | R | 2.05 | 1.54 | 1.68 | 1.16 | | 2.33 | 1.81 | 2.05 | 1.34 |  |
| 10 | L | 0.62 | 0.69 | 0.76 | 0.44 | | 0.95 | 1.03 | 0.92 | 0.69 |  |
|  | R | 0.72 | 0.59 | 0.55 | 0.44 | | 0.81 | 1.12 | 1.01 | 0.69 |  |

Perfusion values from test and retest scans of lower extremity muscle groups in healthy participants (n=10). The data shows the test and retest measurements for the four muscle groups in the lower legs (gastrocnemius (GAS), soleus (SOL), tibialis anterior (TA), and peroneus longus (PL), as well as the four muscle groups in the feet (abductor digiti minimi (ADM), abductor hallucis (AH), flexor digitorum brevis (FDB), and flexor hallucis brevis (FHB)). The values are reported for both the left (L) and right (R) leg.

**S-Table 2: Perfusion values (mL/min/100mL) for test and retest scan of lower legs of all participants using autoradiography**

|  |  | Test | | | |  | Retest | | | | |
| --- | --- | --- | --- | --- | --- | --- | --- | --- | --- | --- | --- |
| Lower legs | | | | | | | | | | | |
| Participant no. | Side | GAS | SOL | TA | PL | | GAS | SOL | TA | PL |  |
| 1 | L | 1.44 | 1.78 | 1.48 | 1.37 | | 1.56 | 1.90 | 1.51 | 1.64 |  |
|  | R | 1.45 | 1.85 | 1.62 | 1.50 | | 1.45 | 1.73 | 1.57 | 1.68 |  |
| 2 | L | 2.44 | 2.81 | 1.55 | 1.65 | | 2.59 | 2.65 | 1.72 | 2.14 |  |
|  | R | 2.51 | 3.39 | 1.62 | 1.98 | | 2.12 | 3.17 | 1.78 | 2.14 |  |
| 3 | L | 1.67 | 2.37 | 1.37 | 1.19 | | 1.64 | 2.64 | 1.26 | 1.25 |  |
|  | R | 1.67 | 1.95 | 1.44 | 1.55 | | 1.89 | 2.55 | 1.51 | 1.50 |  |
| 4 | L | 1.65 | 2.25 | 2.35 | 2.30 | | 1.77 | 2.69 | 2.48 | 2.14 |  |
|  | R | 1.52 | 2.02 | 2.77 | 1.85 | | 1.40 | 2.05 | 3.42 | 1.97 |  |
| 5 | L | 4.00 | 4.54 | 3.47 | 3.77 | | 4.20 | 4.50 | 3.48 | 4.31 |  |
|  | R | 4.17 | 5.07 | 3.22 | 4.45 | | 4.30 | 5.38 | 3.57 | 4.69 |  |
| 6 | L | 2.29 | 2.47 | 2.01 | 1.61 | | 1.85 | 1.80 | 1.85 | 1.32 |  |
|  | R | 2.42 | 3.12 | 1.72 | 2.05 | | 1.86 | 1.90 | 1.59 | 1.45 |  |
| 7 | L | 1.94 | 1.98 | 1.86 | 1.75 | | 2.11 | 1.70 | 1.85 | 1.85 |  |
|  | R | 2.12 | 2.44 | 1.76 | 2.62 | | 2.34 | 2.30 | 1.72 | 2.13 |  |
| 8 | L | 2.15 | 2.56 | 1.26 | 1.47 | | 2.55 | 2.67 | 1.18 | 1.48 |  |
|  | R | 2.27 | 2.71 | 1.60 | 1.89 | | 1.97 | 2.31 | 1.46 | 1.76 |  |
| 9 | L | 1.26 | 1.75 | 1.72 | 1.51 | | 1.72 | 2.59 | 1.67 | 1.55 |  |
|  | R | 2.18 | 2.32 | 1.93 | 1.57 | | 3.01 | 3.84 | 1.92 | 1.88 |  |
| 10 | L | 2.49 | 3.33 | 1.77 | 2.30 | | 2.40 | 2.58 | 1.73 | 2.23 |  |
|  | R | 2.64 | 3.20 | 1.96 | 2.28 | | 2.42 | 2.45 | 1.64 | 2.20 |  |
| Feet | | | | | | | | | | | |
|  |  | ADM | AH | FDB | FHB | | ADM | AH | FDB | FHB |  |
| 1 | L | 0.49 | 0.55 | 0.58 | 0.47 | | 0.68 | 0.53 | 0.77 | 0.44 |  |
|  | R | 0.65 | 0.90 | 1.05 | 0.68 | | 0.82 | 0.88 | 1.06 | 0.64 |  |
| 2 | L | 1.87 | 1.40 | 1.76 | 1.09 | | 1.94 | 1.32 | 1.99 | 1.69 |  |
|  | R | 1.36 | 0.90 | 0.91 | 0.78 | | 1.76 | 1.00 | 1.94 | 0.93 |  |
| 3 | L | 0.56 | 0.56 | 0.56 | 0.74 | | 0.47 | 0.49 | 0.52 | 0.61 |  |
|  | R | 0.56 | 0.98 | 0.72 | 0.80 | | 0.53 | 0.64 | 0.56 | 0.52 |  |
| 4 | L | 3.41 | 1.77 | 1.91 | 1.57 | | 2.98 | 2.25 | 1.82 | 2.12 |  |
|  | R | 2.99 | 1.35 | 2.04 | 2.29 | | 2.70 | 1.46 | 2.00 | 2.28 |  |
| 5 | L | 1.56 | 0.92 | 1.56 | 1.15 | | 1.77 | 1.39 | 1.35 | 1.95 |  |
|  | R | 0.90 | 0.84 | 0.90 | 0.73 | | 1.63 | 0.79 | 1.16 | 0.89 |  |
| 6 | L | 0.73 | 1.00 | 1.51 | 1.35 | | 0.57 | 0.83 | 1.08 | 1.35 |  |
|  | R | 0.41 | 1.09 | 1.15 | 1.13 | | 0.51 | 0.94 | 0.89 | 0.92 |  |
| 7 | L | 0.59 | 0.99 | 0.99 | 0.64 | | 0.56 | 0.80 | 0.83 | 0.74 |  |
|  | R | 0.76 | 1.17 | 1.09 | 0.89 | | 0.73 | 0.81 | 1.02 | 0.70 |  |
| 8 | L | 1.29 | 1.30 | 1.89 | 1.33 | | 0.99 | 1.48 | 1.30 | 1.37 |  |
|  | R | 1.35 | 1.51 | 1.74 | 1.34 | | 1.08 | 1.49 | 1.42 | 1.38 |  |
| 9 | L | 1.73 | 1.15 | 1.72 | 0.80 | | 1.93 | 1.29 | 1.46 | 0.79 |  |
|  | R | 2.10 | 1.64 | 1.82 | 1.09 | | 2.22 | 1.88 | 1.97 | 1.31 |  |
| 10 | L | 0.54 | 0.73 | 0.82 | 0.46 | | 0.80 | 0.96 | 0.84 | 0.64 |  |
|  | R | 0.63 | 0.61 | 0.58 | 0.45 | | 0.72 | 0.93 | 0.97 | 0.64 |  |

Perfusion values from test and retest scans of lower extremity muscle groups in healthy participants (n=10). The data shows the test and retest measurements for the four muscle groups in the lower legs (gastrocnemius (GAS), soleus (SOL), tibialis anterior (TA), and peroneus longus (PL), as well as the four muscle groups in the feet (abductor digiti minimi (ADM), abductor hallucis (AH), flexor digitorum brevis (FDB), and flexor hallucis brevis (FHB)). The values are reported for both the left (L) and right (R) leg.

**S-Table 3: Repeatability of intra- and inter leg/foot perfusion ratio.**

|  | ICC | RPC | ICC | RPC |
| --- | --- | --- | --- | --- |
|  | Lower legs | | Feet | |
| Intra-ratio | 0.85 | 29% | 0.72 | 47% |
| Inter-ratio | 0.71 | 25% | 0.66 | 55% |

ICC: intraclass coefficient, RPC: repeatability coefficient

**S-Table 4: Sample size estimation for increase in perfusion**

|  | Sample Size | |
| --- | --- | --- |
|  | Lower Legs | Feet |
| 25% | 10 | 18 |
| 50% | 5 | 7 |
| 75% | 4 | 5 |

**
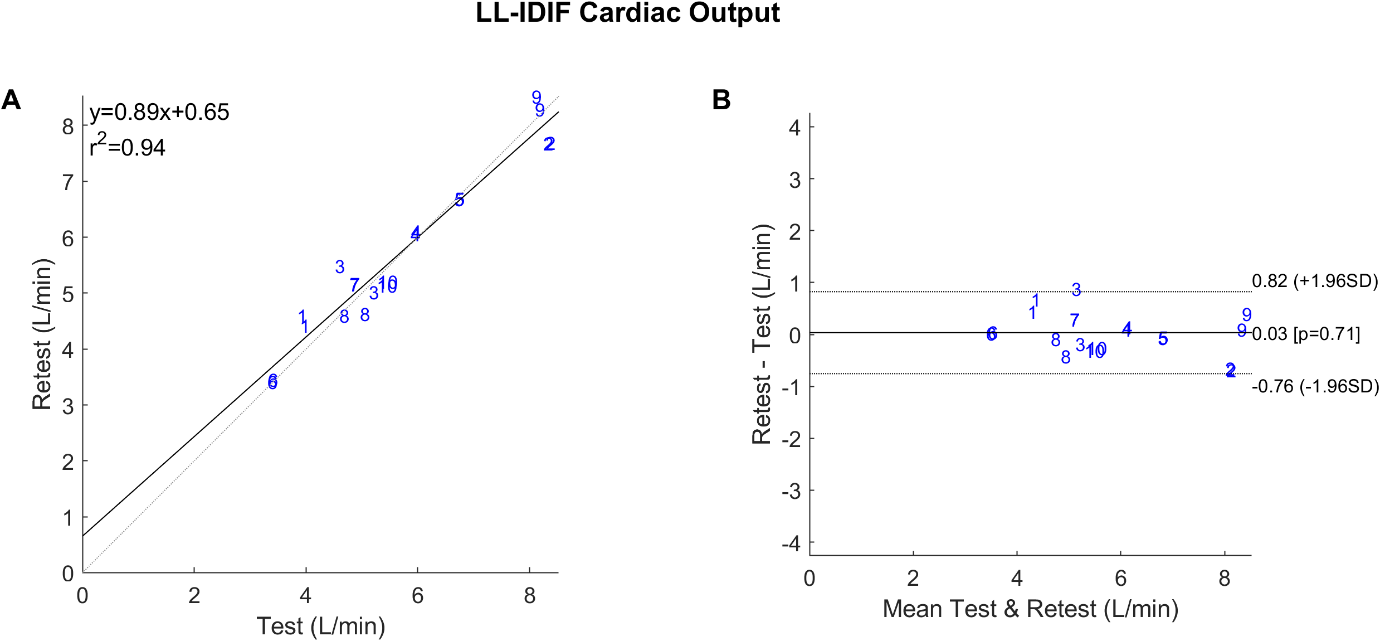
**

**S-Figure 1.** Correlation (A) and Bland-Altman (B) plots demonstrating the repeatability of the Lower Leg IDIF. Plot (A) compares the forward cardiac output calculated from the area under the first pass curve of the delay and dispersion corrected IDIF between test and retest scans for each participant. The blue numbers represent participant identification. Since an IDIF is produced for each leg, each participant is represented twice (n=20). The dashed line represents the line of identity, and the solid line represents the linear fit. Plot (B) shows the mean difference between retest and test scan measurements, with dashed lines indicating the 95% upper and lower limits of agreement.


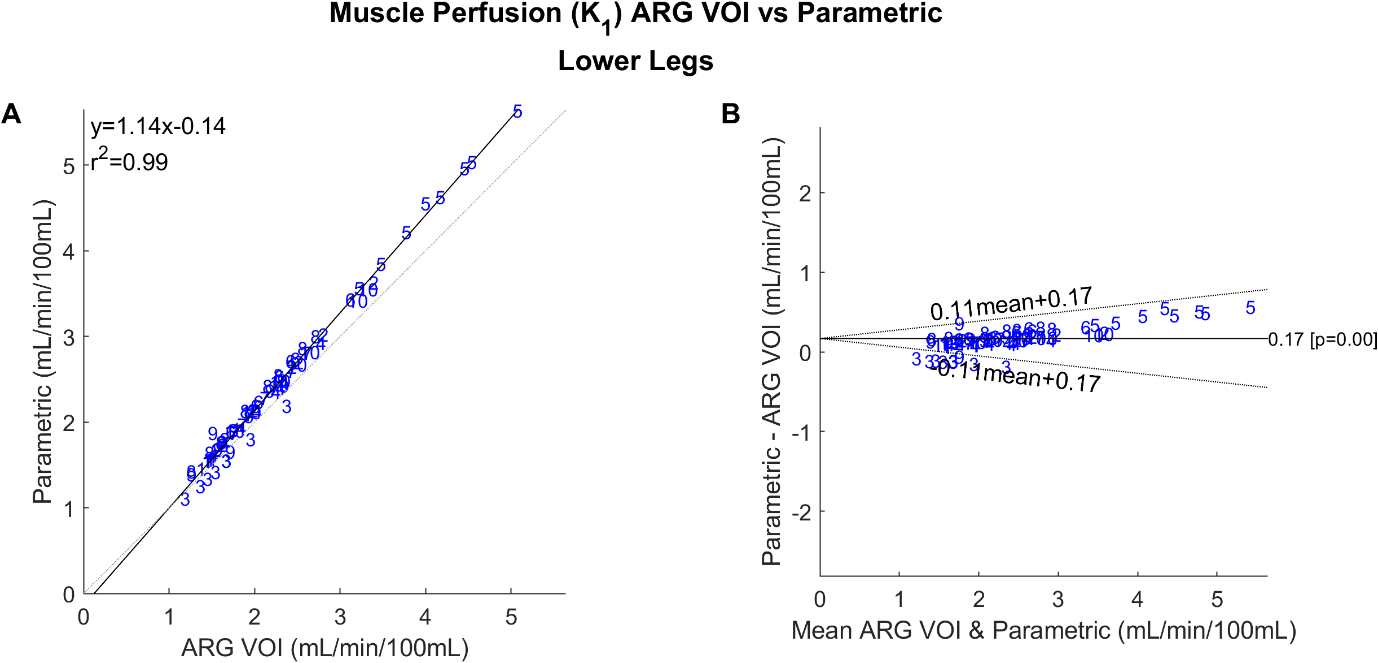

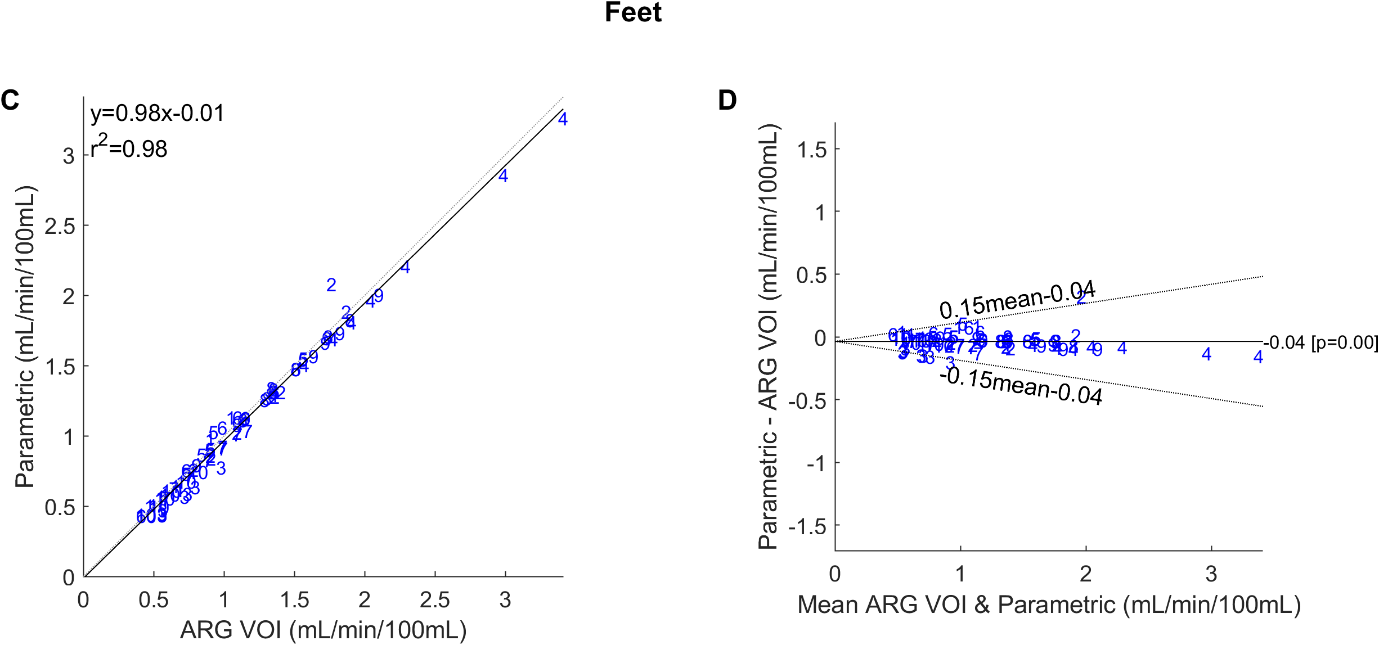


**S-Figure 2:** Correlation plots (A and C) and Bland-Altman plots (B and D) comparing the ARG and parametric image methods in lower legs (A and B) and feet (C and D). The blue numbers represent participant identification. The analysis includes perfusion values from all participants, both the left and right leg and foot (n=80). The correlation plots compare the K_1_ values estimated from the methods, and include coefficient of determination (r^2^), and the linear equation. The dashed lines represent the lines of identity, while the solid lines represent the linear fit. The Bland-Altman plots display the mean difference between the K_1_ measurements from the ARG and 1TCM method, with the dashed lines representing the 95% upper and lower limits of agreement.


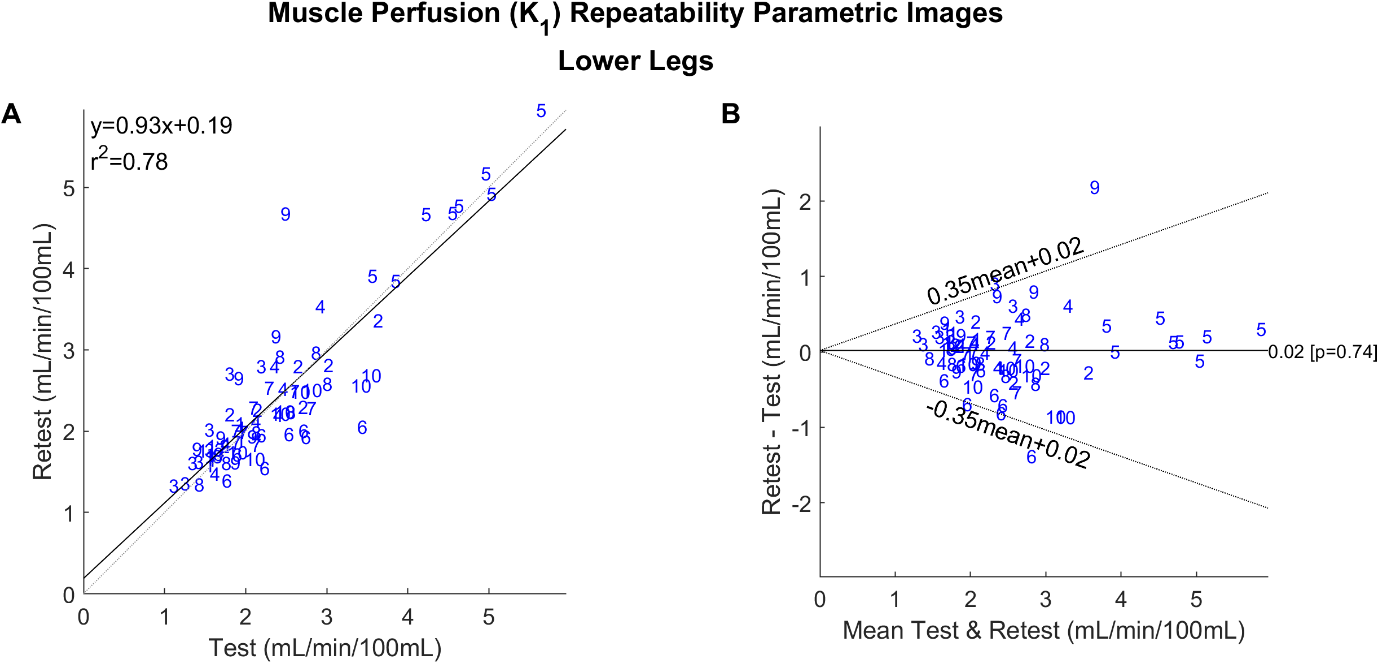

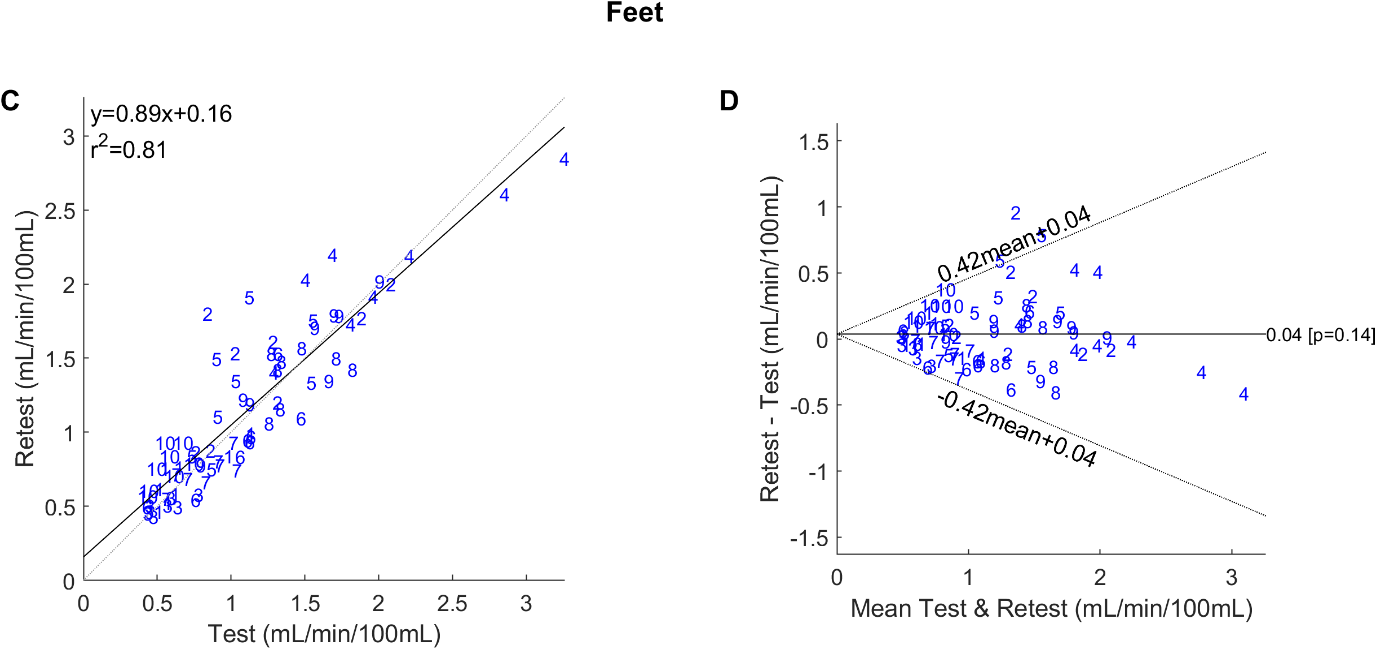


**S-Figure 3:** Correlation plots (A and C) and Bland-Altman plots (B and D) demonstrating the repeatability of parametric images in lower legs (A and B) and feet (C and D). The blue numbers represent participant identification. The analysis includes perfusion values from all participants, both the left and right leg and foot (n=80). The correlation plots compare the K_1_ values estimated from the methods, and include coefficient of determination (r^2^), and the linear equation. The dashed lines represent the lines of identity, while the solid lines represent the linear fit. The Bland-Altman plots display the mean difference between the K_1_ measurements from the ARG and 1TCM method, with the dashed lines representing the 95% upper and lower limits of agreement.
